# HSP60 and HSP10 depletion provoke distinct biological responses

**DOI:** 10.1098/rstb.2026.0194

**Published:** 2026-10-01

**Authors:** Hila Ben-David, Shani Gabbay, Sufa Sued-Hendrickson, Linor Cohen, Liron Levin, Nili Tickotsky Moskovitz, Ifat Abramovich, Shiran Dror, Yariv Brotman, Shai Kaluski-Kopach, Evelina Nikelshparg, Anat Ben-Zvi, Barak Rotblat

**Affiliations:** ^1^ Department of Life Sciences, Ben-Gurion University of the Negev, Be'er Sheva, 8410501, Israel; ^2^ Ilse Katz Institute (IKI) for Nanoscale Science and Technology, Ben-Gurion University of the Negev, Be'er Sheva, 8410501, Israel; ^3^ Perlmutter Metabolomics Center, Technion Israel Institute of Technology, Haifa 32000, Israel; ^4^ School of Plant Sciences and Food Security, The George S. Wise Faculty of Life Sciences, Tel Aviv University, Tel Aviv-Yafo 69978, Israel

**Keywords:** mitochondria, chaperonin, development, cancer, mitochondrial UPR

## Abstract

The mitochondrial chaperone and co-chaperone HSP60/HSP10 (HSPD1/ HSPE1), the mammalian homologues of bacterial GroEL/GroES, form a barrel-and-lid complex to fold newly imported or misfolded mitochondrial proteins. Several lines of evidence suggest that HSPD1 and HSPE1 have diverged in function. HSPD1 and HSPE1 are found in different clusters in the ecological network of gene expression in cancer. MTHFD2 was demonstrated to be an endogenous client protein of HSPD1 that does not require HSPE1 for its folding. Newly imported proteins show differential dependencies on HSP60 and HSP10 in isolated yeast mitochondria. Finally, HSPE1 was found to regulate a mitochondrial GTPase and modulate amyloid fibril formation independently of HSPD1. Nevertheless, the extent to which HSPD1 and HSPE1 have diverged in function has not been explored. Here, we show divergent transcriptomic and metabolic responses to the depletion of HSP60 versus HSP10 in cancer cells, and divergent activation of the mitochondrial unfolded protein response across tissues in *Caenorhabditis elegans*. Importantly, responses to HSP10 depletion were not nested within those of HSP60 depletion. We speculate that HSPE1 has diverged from HSPD1 and may have additional new cellular functions.

This article is part of the Theo Murphy meeting issue ‘ProteostaSys: a systems view of proteostasis’.

## 1. Introduction

Proteostasis is maintained by a protein quality control network composed of chaperones and co-chaperones, as well as the protein degradation machinery. When the proteostasis network fails, misfolded and damaged proteins accumulate, leading to pathological outcomes. Evidence supports the idea that in metazoans, supply and demand shape the expression of the quality control network to match the needs of the tissue-specific proteome at particular developmental stages [1–3]. This model aligns with the tissue specificity of pathologies caused by germline mutations that affect protein folding [4], such as polyglutamine repeat expansions or mutations in chaperones [5,6]. In addition, this model posits that specific proteins depend on specific chaperones for folding. Which proteins depend on which chaperones for folding, in general and specifically in a given tissue, is largely unknown.

The majority of mitochondrial proteins are encoded in the nuclear genome, translated in the cytosol and imported to the mitochondria as linear peptides, after which they are folded by mitochondrial chaperones [7]. It can be argued that a gene encoding a protein that depends on a specific chaperone will evolve coordinated expression with that chaperone; therefore, co-expression might indicate that the protein depends on the coexpressed chaperone. Using gene expression data from multiple tumour types and ecological network analysis, a mitochondrial chaperone–protein co-expression network was constructed and found to comprise two major modules [8,9]. These findings show that not all mitochondrial proteins and chaperones are coexpressed and suggest that different chaperones and co-chaperones support the folding of distinct sets of proteins. Surprisingly, HSPD1 and HSPE1, members of the same chaperone complex that share a promoter and are coexpressed, are found in different network clusters [8,9], suggesting that they may have divergent functions.

HSPD1 (HSP60) and HSPE1 (HSP10), the human homologues of the bacterial GroEL/GroES, are conserved chaperones. Together, they form a barrel-and-lid complex that encapsulates misfolded proteins, supporting their refolding in a secluded environment [10–12]. A common assumption is that HSPE1 is indispensable for HSPD1 function [13–16]. Nevertheless, human and bacterial HSP60 were shown to fold some model misfolded proteins independently of HSP10 *in vitro* [17–19]. Using mitochondria isolated from yeast, it was found that the solubility of a number of newly imported proteins was HSP60-dependent and HSP10-independent, and *vice versa* [20,21]. Importantly, HSP10-dependent proteins were not all nested within the list of HSP60-dependent proteins, suggesting that HSP10 can also function independently of HSP60. In human cells, HSPE1, but not HSPD1, was found to promote the function of OPA1, a mitochondrial GTPase [22,23]. Furthermore, human, mouse and *Drosophila* HSP10 were shown to modulate α-synuclein and Aβ1–42 amyloid fibril formation independently of HSPD1 [24–26]. These data suggest that HSPE1 may have evolved divergent functions.

Methylenetetrahydrofolate dehydrogenase 2 (MTHFD2) is an endogenous HSPD1 client protein [27]. Interestingly, HSPD1 folds MTHFD2 independently of HSPE1 *in vitro* and in cells, providing proof of concept that HSPD1 can fold an endogenous substrate independently of HSPE1 [27]. Nevertheless, the extent to which HSPD1 and HSPE1 interact with distinct cellular pathways and exhibit divergent functions across tissues remains unknown.

Here, we found that in cancer cells, depletion of HSPD1 elicits very different cellular and stress responses at the transcriptomic and metabolic levels compared with HSPE1 depletion. Furthermore, using *Caenorhabditis elegans*, we found that the mitochondrial unfolded protein response (mtUPR) is triggered in different tissues in response to HSP60 versus HSP10 knockout (KO). Together, these data support the model predicting that, in addition to their classical roles, HSPD1 and HSPE1 have evolved divergent functions in metazoans.

## 2. Results

### (a) Divergent transcriptional response to HSPD1 and HSPE1 knockdown

To determine the extent to which the biological functions of HSPD1 and HSPE1 have diverged, we took a systems approach. We hypothesized that if HSPD1 and HSPE1 have distinct functions or assist in the folding of different sets of proteins, cells will respond very differently to their knockdown (KD). However, if HSPD1 and HSPE1 primarily work together to fold the same set of proteins, we expect that depleting either protein will lead to the aggregation and degradation of a similar set of proteins. In this case, the cells would likely respond to these changes in a similar manner, reflected in comparable transcriptional changes.

To test the hypothesis that HSPD1 and HSPE1 interact with distinct cellular pathways, we used either HSPD1 or HSPE1 KD (two independent short hairpin RNAs (shRNAs) for each KD and a scrambled control) in A549 and U251, lung and brain cancer cells, respectively (with KD efficiency >70% in the RNA-seq data). We compared the cellular response to HSPD1 and HSPE1 KD using RNA-seq. We found substantial differences in the transcriptomes of HSPD1 KD and HSPE1 KD A549 cells (figure 1A; supplementary material, table S1). More than 700 transcripts were up- or downregulated following HSPD1 or HSPE1 KD, of which <10% were shared between the chaperone and its co-chaperone (figure 1B; supplementary material, table S1). These differences were also reflected in the biological categories enriched in the list of up- or downregulated transcripts in HSPD1 and HSPE1 KD cells (figure 1C). For example, categories related to oxidative stress, such as glutathione metabolic processes, were enriched in the list of downregulated transcripts in HSPD1 KD but not HSPE1 KD cells (figure 1C). Conversely, categories related to RNA processing were enriched in the list of downregulated transcripts in HSPE1 KD but not HSPD1 KD cells (figure 1C).

**Figure 1.**
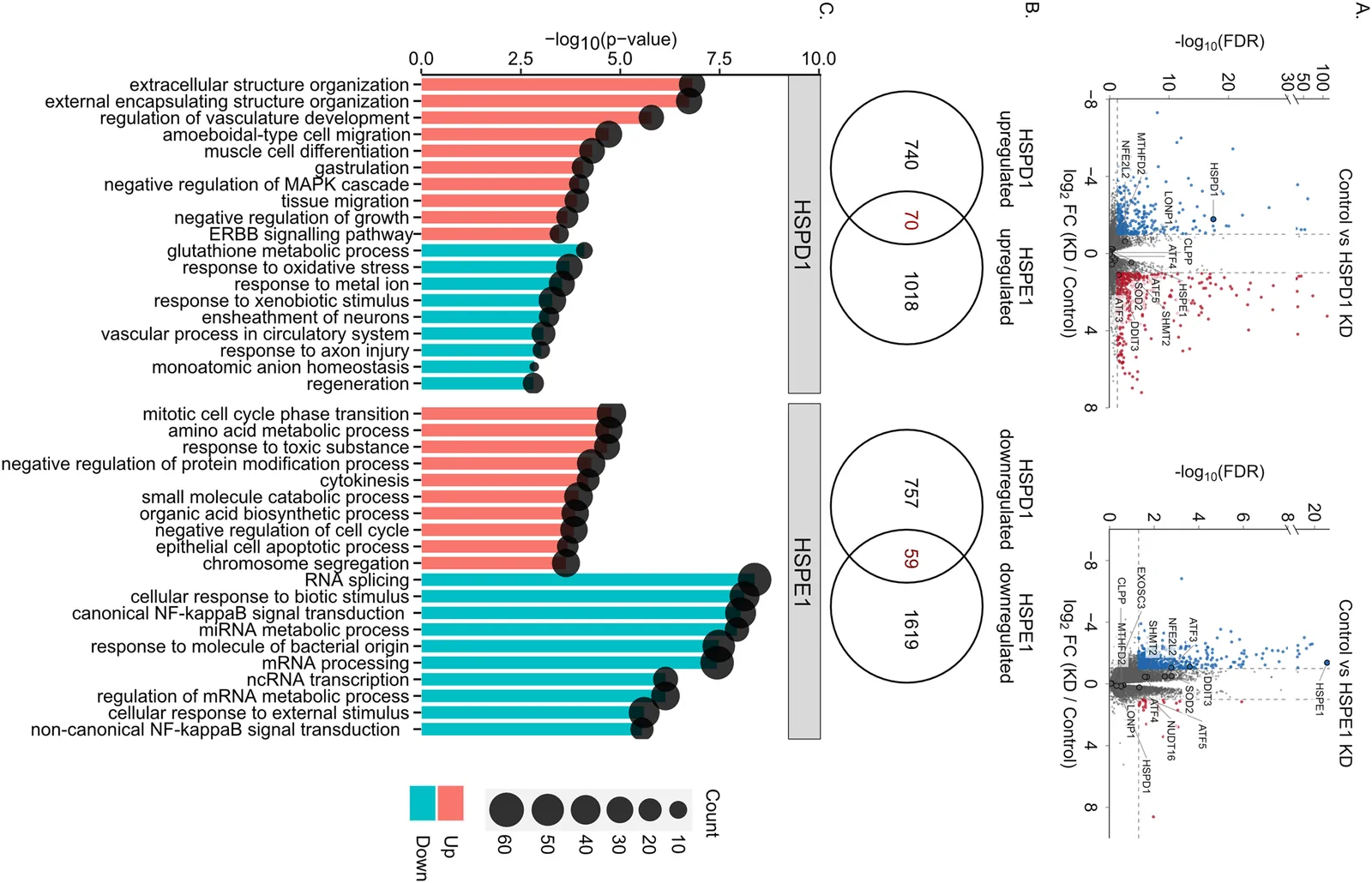
The impact of HSPD1 versus HSPE1 KD on gene expression. (A) Gene expression in HSPD1 or HSPE1 knockdown (KD) versus control A549 cells was measured using RNA-seq. shCONTOL, *n* = 4; shHSPD1#1, *n* = 4; shHSPD1#2, *n* = 4; pooling the two KD cell lines together. Data are shown using a volcano plot. Depletion of HSPD1 and HSPE1 in the respective cells is indicated in the volcano plot. (B) Venn diagram of the number of significantly up- or downregulated transcripts in HSPD1 KD and HSPE1 KD cells. (C) Pathways enriched in transcripts upregulated or downregulated by HSPD1 and HSPE1 KD. FC, fold change; FDR, false discovery rate; miRNA, microRNA; ncRNA, non-coding RNA.

The mtUPR is a transcriptional programme triggered when mitochondrial folding demands exceed their folding capacity [28]. Focusing on stress response transcripts, we also found no overlap between the HSPD1 KD and HSPE1 KD upregulated genes. HSPD1 KD induced *SIRT3*, *EIF2AK4*, *SOD2*, *DNAJC1*, *HSP90AA1*, *HSPB8*, *JUN*, *P53* and *MDM2*, while HSPE1 KD induced *DNAJC22*, *CAT*, *GPX2*, *HMOX1 RAD51AP1*, *RAD54L* and *TXNRD3*. These differences were also found specifically in mtUPR transcripts. We did not find upregulation of classic mtUPR genes, including *ATF4*, *ATF5*, *DDIT3* (*CHOP*), *HSPD1*, *HSPE1*, *CLPP* and *LONP1* [29–31] in HSPD1 or HSPE1 KD cells. Surprisingly, we found reduced expression of some stress response genes, such as *NFE2L2* (*NRF2*) and its target *HMOX1*, in HSPD1 KD cells. In addition, some mtUPR genes were downregulated in HSPE1 KD cells, including *ATF4* and *CHOP*. An interesting case is *SOD2* [32], which was upregulated in HSPD1 KD cells and downregulated in HSPE1 KD cells.

We validated these findings in U251 cells (supplementary material, figure S1A and table S1) and found that, as in A549 cells, there were profound transcriptional changes following HSPD1 KD or HSPE1 KD. More than 700 transcripts were affected by the KD, with 17 transcripts showing changes in expression under both HSPD1 and HSPE1 KD (supplementary material, figure S1B). In line with results obtained with A549 cells, the biological categories enriched in the lists of up- or downregulated transcripts in HSPD1 and HSPE1 KD cells were very different. For example, transcripts related to actin filament organization were upregulated in HSPD1 KD cells, while cytoplasmic translation was upregulated in HSPE1 KD cells (supplementary material, figure S1).

Stress genes, including *AFG3L2*, *BAK1*, *HSPB8*, *MDM2* and *RAD9B*, were upregulated in HSPD1 KD but not HSPE1 KD cells. By contrast, mtUPR genes, including *ATF4*, *ATF6*, *LONP1*, *ASNS* and *CHOP*, were induced in HSPE1 KD cells but not HSPD1 KD cells. These data show that the transcriptional response to KD of either HSPD1 or HSPE1 varies, consistent with the hypothesis that they cater to the folding of distinct protein groups.

Although the divergent response between HSPD1 KD and HSPE1 KD is maintained in A549 and U251 cells, the two cell lines respond differently to similar perturbations. mtUPR, for example, HSPE1 KD, induced *ATF4* and *CHOP* in U251 but reduced *ATF4* and *CHOP* in A549 cells. Interestingly, HSPD1 KD did not induce the mtUPR in either cell line, suggesting that it may trigger mtUPR in a specific biological context not recapitulated in the cancer cells we used. Another example is *SOD2*, which was upregulated in HSPD1 KD A549 cells but not in U251 cells. *SOD2* was shown to be upregulated in the brains but not the spinal cords of *Hspd1* heterozygous mice [32]. Such context may relate to the tissue dependence on HSPD1 targets in a particular cellular context.

### (b) Divergent metabolic response to HSPD1 and HSPE1 knockdown

The composition and function of mitochondria have profound effects on cellular metabolism. We reasoned that if the major function of HSPD1 and HSPE1 is to work together to fold proteins, perturbations of either will lead to the collapse of a similar set of mitochondrial proteins, and cells will respond by adjusting their metabolism in a similar fashion. However, if HSPD1 and HSPE1 have diverged in function, we expect that depleting either HSPD1 or HSPE1 will cause the collapse of proteins involved in distinct metabolic pathways and, as a result, each chaperone's depletion will affect cellular metabolism differently.

To this end, we used targeted metabolomics analysis to identify metabolic changes in response to HSPD1 or HSPE1 KD in A549 cells growing in human plasma-like media. We designated metabolites that were significantly decreased or increased in the two KD cell lines compared with control cells as positive hits (one-way ANOVA, *p* < 0.05 after Bonferroni multiple-testing correction). Of 216 metabolites analysed, two were downregulated and two upregulated in HSPD1 KD cells but not in HSPE1 KD cells (figure 2A; supplementary material, table S2). Two metabolites were upregulated in both HSPD1 KD and HSPE1 KD cells (figure 2B). Eight metabolites were upregulated in HSPE1 KD but not in HSPD1 KD cells (figure 2C). These findings corroborate that the metabolic profiles of cells undergo distinct alterations following the knockdown of HSPD1 compared with HSPE1.

**Figure 2.**
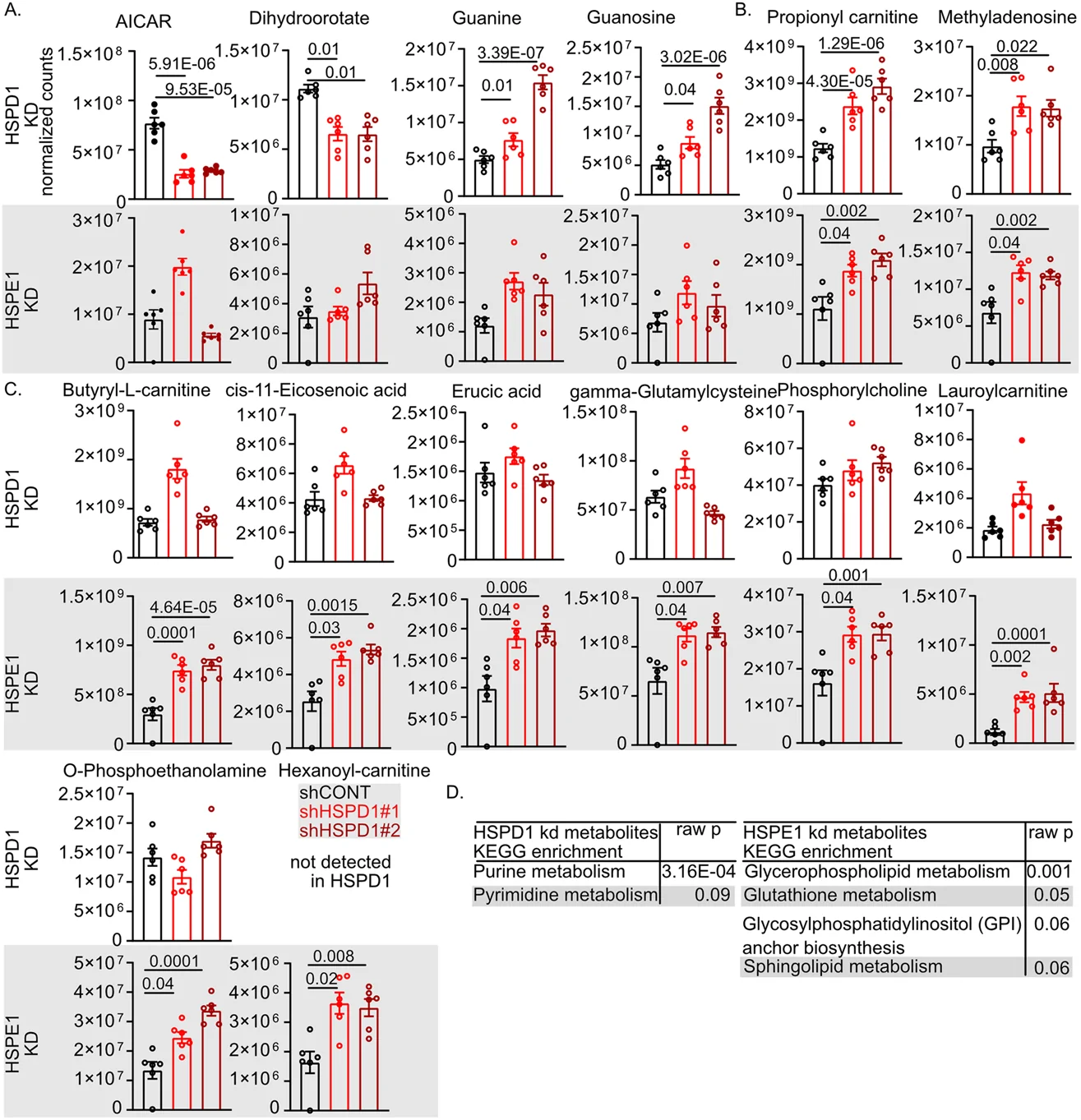
Metabolites in HSPD1 knockdown (KD) and control cells. A549 control and HSPD1 or HSPE1 KD cells were analysed by targeted metabolomics. The graph shows mean counts; error bars show s.e.m.; black are control cells, red and brown are KD cells. shCONTROL, *n* = 6; shHSPD1#1, *n* = 6; shHSPD1#2, *n* = 6; shHSPE1#1, *n* = 6; shHSPE1#2, *n* = 6; *p*-values were obtained using one-way ANOVA adjusted for multiple testing using Bonferroni correction. In cases where metabolites were significantly changed in the same direction in two KD cell lines (*p* < 0.05), these were considered positive hits. Top rows show the data for HSPD1 KD versus control; below, shaded in grey, are the data for HSPE1 KD versus control. (A) Metabolites significantly affected by HSPD1 KD (same direction in two KD cell lines) but not changed by HSPE1 KD. (B) Metabolites were significantly affected in both HSPD1 KD and HSPE1 KD cells. (C) Metabolites were affected by HSPE1 KD but not by HSPD1 KD. (D) MetaboAnalyst identified enriched metabolic pathways in HSPD1 KD and HSPE1 KD cells. AICAR, 5-aminoimidazole-4-carboxamide ribonucleoside; KEGG, Kyoto Encyclopedia of Genes and Genomes.

We used MetaboAnalyst 6.0 (metaboanalyst.ca) to identify pathways enriched in our list of metabolites significantly affected by HSPD1 and HSPE1 depletion (figure 2D). Purine metabolism was the most significantly enriched pathway in HSPD1 KD cells. By contrast, glycerophospholipid metabolism was found enriched in HSPE1 KD cells (figure 2D). Interestingly, the accumulation of acylcarnitines (butyryl-l-carnitine, hexanoyl carnitine, lauroylcarnitine, propionyl carnitine) in HSPE1 KD cells suggests compromised mitochondrial β-oxidation [33], which is in line with previous metabolic analysis of the consequence of HSPE1 depletion in neurons [34].

We identified purine metabolism as the major pathway affected by HSPD1 depletion, which included decreased 5-aminoimidazole-4-carboxamide ribonucleoside (AICAR) levels and increased free purine levels. Nevertheless, in a parallel publication [27], we found that MTHFD2, a key enzyme in the mitochondrial one-carbon pathway, is eliminated in HSPD1 KD cells, predicting a decrease, rather than an increase, in purines [35] and upregulation, rather than downregulation, of AICAR [36]. AICAR downregulation was found in cases where, in addition to MTHFD2, another one-carbon enzyme, PAICS, was depleted [37]. Using SILAC-based proteomics to identify HSPD1-dependent proteins, we found that the mitochondrial one-carbon enzyme serine hydroxymethyltransferase 2 (SHMT2) was also depleted in HSPD1 KD cells [27]. SHMT2 is upstream to MTHFD2 and is therefore the second downregulated one-carbon enzyme in HSPD1 KD cells.

At the transcriptomic level, we found downregulation of central one-carbon, folate and purine biosynthetic genes *GLDC*, *DHFR* and *ATIC* in HSPD1 KD A549 cells. Because mitochondrial one-carbon supply enzymes (MTHFD2, SHMT2, GLDC, DHFR) and the purine synthesis machinery (ATIC) are downregulated in HSPD1 KD cells, it is reasonable to expect reduced purine synthesis and AICAR depletion. In addition, we found evidence of reduced de novo purine synthesis and increased RNA degradation and salvage, which could explain the increase in free purines but not triphosphate purines. In support, 1-methyladenosine, a product of rRNA or tRNA degradation, was increased in HSPD1 KD cells. Together, these data support reduced one-carbon output in HSPD1 KD cells and are in accord with HSPD1 supporting the one-carbon pathway.

These results show that the metabolic responses to HSPD1 or HSPE1 KD differ markedly in A549 cells, further supporting the divergent biological impacts of the two on cellular functions.

### (c) The mitochondrial stress response is activated in different tissues following HSP60 or HSP10 knockout in *Caenorhabditis elegans*

Our transcriptomic and metabolic data are consistent with the model, indicating that HSP60 and HSP10 support the folding of distinct sets of mitochondrial proteins and thus affect different metabolic pathways and cellular responses. This model predicts that HSP60 and HSP10 could have important functions, resulting in different outputs in different tissue types. To test this hypothesis in a whole organism, we used *C. elegans*. We employed CRISPR KO of either *hsp-60* or *hsp-10* (gene name *Y22D7AL.10*). The two KO strains caused larval arrest at the L2–L3 stage and were thus maintained as heterozygotes. Validated homozygous KO or wild-type (WT) progeny were used for the subsequent experiments.

To test whether HSP60 or HSP10 KO has divergent cellular responses across tissues, we focused on mtUPR activation as a readout of mitochondrial proteostasis. To this end, we crossed worms engineered to harbour green fluorescent protein (GFP) under the *hsp-6* promoter (*C. elegans* HSPA9 orthologue; strain SJ4100), which is activated by the transcription factor ATFS-1, a classic readout of mtUPR [35,36], with our KO heterozygote strains. Experiments were performed using homozygous KO or WT progeny expressing the mtUPR reporter. We chose this model because we assume that if KO of *hsp-60* or *hsp-10* has differential mitochondrial cellular impacts, we will observe distinct GFP expression patterns. In other words, if, in general, HSP-60 and HSP-10 were to work together to fold the same subset of proteins, we would expect that similar tissues would experience mtUPR, and a similar GFP expression pattern would be observed in either *hsp-60* or *hsp-10* KO.

We found *hsp-60* mutants expressed GFP in the gut, while *hsp-10* KO mutants exhibited GFP expression in muscles (figure 3A). In accord, we found decreased motility rates, a readout for muscle function, in *hsp-10* but not in *hsp-60* KO worms (figure 3B). These data support our hypothesis that HSP60 and HSP10 have divergent functions in the whole organism and that such divergent functions are found in nematodes as well as in cancer cell lines.

**Figure 3.**
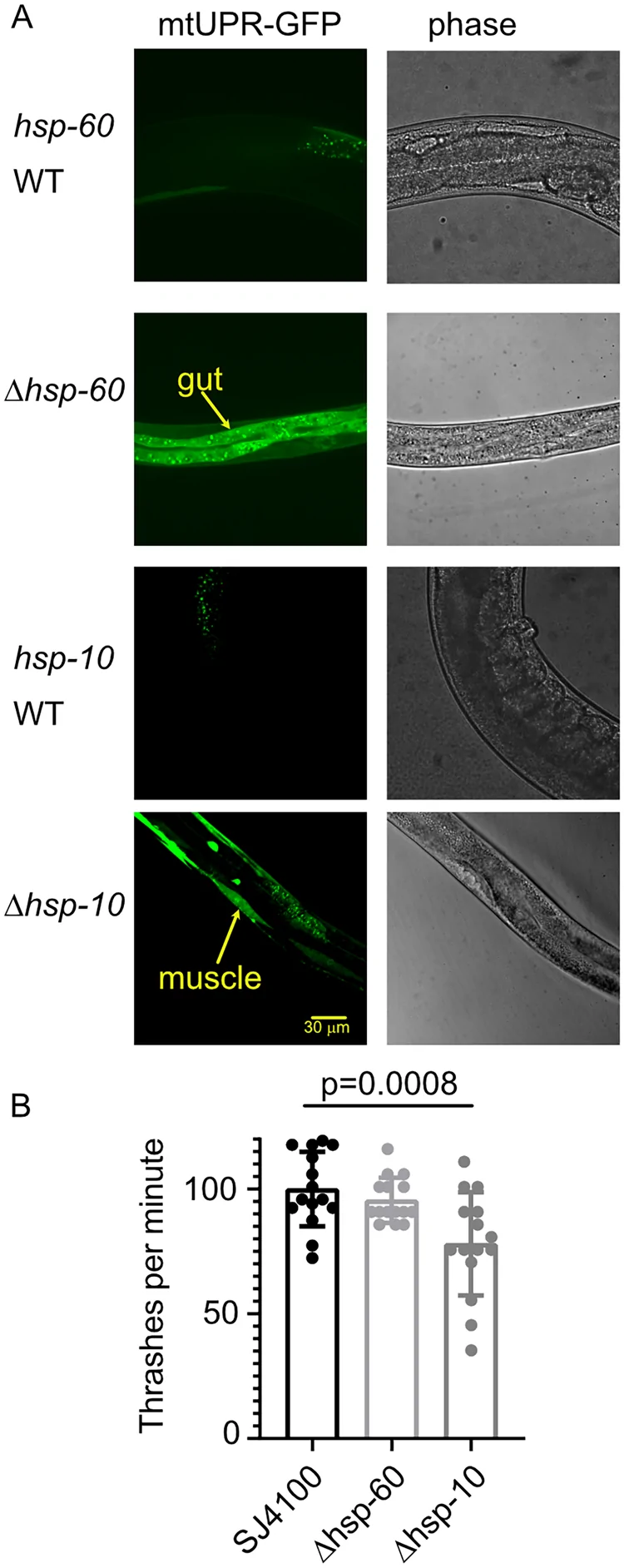
Divergent phenotypes of HSP-60 versus HSP-10 knockout (KO) in *Caenorhabditis elegans*. (A) Representative images of mitochondrial unfolded protein response (mtUPR) activation. Green fluorescent protein (GFP) expression indicates mtUPR activation in homozygous *hsp-60* or *hsp-10* KO versus homozygous wild-type ( WT) progeny of heterozygous *hsp-60* or *hsp-10* worms, respectively. (B) Thrashing was recorded in the indicated homozygous strains. Data are shown as mean ± s.e.m. (*n* = 15). *p*-value was obtained using one-way ANOVA.

## 3. Discussion

The mitochondrial chaperone–client co-expression network in cancer reveals that HSPD1 and HSPE1 cluster in distinct groups, despite their co-expression and shared promoter [8,9]. These data suggest that they evolved to be expressed with different proteins and may, perhaps, be a limiting factor in the folding of a different set of proteins. One explanation for this phenomenon is that HSPD1 can fold some proteins independently of HSPE1, while for others, HSPE1 is essential. In this case, we expect that the response to HSPE1 depletion will be nested within the response to HSPD1 depletion, as we expect that HSPE1-dependent proteins will also be HSPD1-dependent, while some of the HSPD1-dependent proteins will not be HSPE1-dependent. Conversely, HSPE1 may have acquired distinct moonlighting functions beyond its association with HSPD1.

Here, we show that the metabolic and transcriptomic changes triggered by HSPD1 depletion in cancer cells are markedly different from those caused by HSPE1 depletion. Notably, the alterations observed following HSPE1 depletion are not nested within those resulting from HSPD1 depletion. The simplest interpretation of these results is that HSPE1 has important cellular functions that are independent of HSPD1. These functions may be related to protein folding and proteostasis or to other, more divergent functions.

HSPE1 was shown to modulate α-synuclein and Aβ1–42 amyloid fibril formation independently of HSPD1 [24–26], supporting an independent protein folding function for HSPE1. Specifically, HSPE1 but not HSPD1 levels were reduced in samples from Parkinson's disease patients [24]. HSPE1 interacted *in vivo* and *in vitro* with α-synuclein and was sequestered in the cytosol of cells expressing α-synuclein [24,25]. Moreover, HSPE1 inhibited WT and mutant α-synuclein fibril formation but promoted fibril formation at sub-stoichiometric concentrations [25]. HSPE1 depletion also resulted in proteolytic cleavage and inactivation of OPA1 by the stress-activated metalloprotease OMA1, independently of HSPD1 [23]. How HSPE1 protects OPA1 and whether this function is linked to protein folding remains open. However, the concept that chaperones have biological functions beyond protein folding is not new. For example, a small heat shock protein (sHSP) binds to actin to regulate cell migration [37,38]. It was also found that HSP70 specifically binds the transcription factor HSF1 to regulate its activity [39] and can also regulate the stability of the transcription factors SOX10 and GATA-1, which regulate oligodendrocyte and erythrocyte differentiation, respectively [1,40]. Chaperones were also shown to play signalling roles in the extracellular space [41] and when refluxed from cellular organelles into the cytosol [42]. We therefore speculate that HSPE1 has evolved HSPD1-independent functions, which may explain the very different responses to HSPD1 versus HSPE1 depletion.

Different tissues express distinct sets of proteins and depend on distinct chaperones [1,4]. This is consistent with findings in *Hspd1* heterozygous mice, in which distinct gene programmes are activated in different anatomical regions [43]. Using *C. elegans* engineered to express GFP in cells that activate mtUPR, we found that HSP60 depletion triggered mtUPR in the gut, whereas HSP10 depletion triggered it in muscles. Interestingly, the mtUPR activation in HSP10 mutant worms was not nested within the response in the HSP60 mutants. In accord with our findings in cancer cells, these data support that HSP10 has an HSP60-independent function, as HSP60 KO did not result in mtUPR activation in muscles.

A possible explanation for these results is that, in muscle mitochondria, other chaperones can compensate for the absence of HSP60 but not for HSP10, or that, in this specific context, HSP60 acts as a dominant negative when HSP10 is unavailable. This may be similar to the case where overexpression of HSPB3 drives muscle differentiation, as HSPB3 binds to and inhibits lamin-B receptors in muscle [44]. We also found mtUPR activation in the gut of HSP60-mutant worms but not HSP10 mutants. These findings suggest that gut mitochondria exhibit greater tolerance to HSP10 depletion than to HSP60 depletion. It is therefore plausible that HSP60 is essential and sufficient for proper protein folding in gut mitochondria. Indeed, we found that HSPD1 can fold the mitochondrial enzyme MTHFD2 independently of HSPE1 *in vitro* and in cells [27], which is consistent with MTHFD2 being part of the HSPD1 cluster rather than the HSPE1 cluster in the mitochondrial chaperone–protein co-expression network [9]. Alternatively, HSP60 may have evolved additional biological functions beyond protein folding, which could explain mtUPR activation in the gut of HSP60 KO worms.

In the tested cancer cells, we found that only HSPE1 KD in U251 cells triggered mtUPR. Why A549 cells do not trigger mtUPR upon KD of HSPD1 or HSPE1 is not clear, but may be linked to lower expression of oxidative phosphorylation (OXPHOS)-related genes, including *NDUFA13*, *HK1*, *HK2* and *COX18*, and higher expression of *LDHA* (supplementary material, figure S2), suggesting they are less dependent on mitochondria to begin with. There is also higher *CHOP* expression in U251 cells, suggesting they are poised to induce mtUPR.

The differential effects of HSPD1 KD across different biological contexts reported here are consistent with previous studies of HSPD1 depletion in zebrafish larvae, in cells expressing mutant HSPD1, and in fibroblasts from patients harbouring pathogenic *HSPD1* mutations [39]. For example, the integrated stress response (ISR) and mtUPR genes were differentially upregulated across the systems. In HEK293 cells expressing the HSP60-D423A mutation, *ATF3*, *ATF4*, *MTHFD2* and *SHMT2* were upregulated, whereas in *hspd1* KO larvae, only *atf3* was induced. In patient fibroblasts, there were no clear mtUPR or ISR upregulated transcripts [39], as was the case in HSPD1 KD A549 and U251 cells. In *C. elegans*, mtUPR was activated in response to *hsp-60* or *hsp-10* in specific cell types. The most consistent cellular response to HSPD1 mutation or deficiency across different biological models was the apparent downregulation of the one-carbon pathway, as evidenced by increased serine and glycine and reduced MTHFD2 and SHMT2 protein levels. These results are consistent with our data showing that HSPD1 plays a key role in the one-carbon pathway [27].

Although evidence suggests that HSPE1 performs functions independently of HSPD1, a primary limitation of this research is that the specific nature of these functions remains unidentified. Nonetheless, our findings demonstrate that HSPD1 and HSPE1 engage with divergent pathways in cancer cells. Furthermore, their respective depletions induce the mtUPR in distinct tissues of *C. elegans*, supporting that HSP10 has evolved HSP60-independent functions.

## 4. Methods

Please see supplementary material for methods.

## Supplementary Material



## Data Availability

RNA-seq data were deposited in the GSE database: https://www.ncbi.nlm.nih.gov/CBBresearch/Lu/Demo/GeneAgent/?id=4CB78AE38E844FA1DDF9; the mitochondrial chaperone HSPD1 folds MTHFD2 independently of its co-chaperone HSPE1 [A549HSPD1 KD]: GSE314357; the mitochondrial chaperone HSPD1 folds MTHFD2 independently of its co-chaperone HSPE1 [U251KD E1]: GSE303582. Supplementary material is available online [45].

## Theme

One contribution of 20 to a Theo Murphy meeting issue ‘ProteostaSys: a systems view of proteostasis’.
