# Supplementary material for: HSP60 and HSP10 depletion provoke distinct biological responses
Source: Philos Trans R Soc Lond B Biol Sci. 2026 Oct 1;381(1960):20260194. doi: 10.1098/rstb.2026.0194 (PMC13628081; doi:10.1098/rstb.2026.0194)
Supplement: Supplementary file 1 [file rstb.2026.0194.zip › rstb.2026.0194/rstb20260194_si_001.pdf]

Supplementary information

Supplementary Figures and Methods

HSP60 and HSP10 depletion provoke distinct biological responses

Hila Ben-David*1, Shani Gabbay*1, Sufa Sued-Hendrickson*1, Linor Cohen1, Liron Levin2, Nili Tickotsky Moskovitz2, Ifat Abramovich3, Shiran Dror2, Yariv Brotman1, Shai Kaluski-Kopatch1, Evelina Nikelshparg1, Anat Ben-Zvi1, Barak Rotblat1#

* equal contribution

# Corresponding

AFFILIATIONS

1 Department of Life Sciences, Ben-Gurion University of the Negev, Beer-Sheva, Israel

2 Ilse Katz Institute (IKI) for Nanoscale Science and Technology, Ben-Gurion University of the Negev, Beer Sheva, Israel

3 Laura and Isaac Perlmutter Metabolomics Center, B. Rappaport Faculty of Medicine, Technion-Israel Institute of Technology, Haifa, Israel.

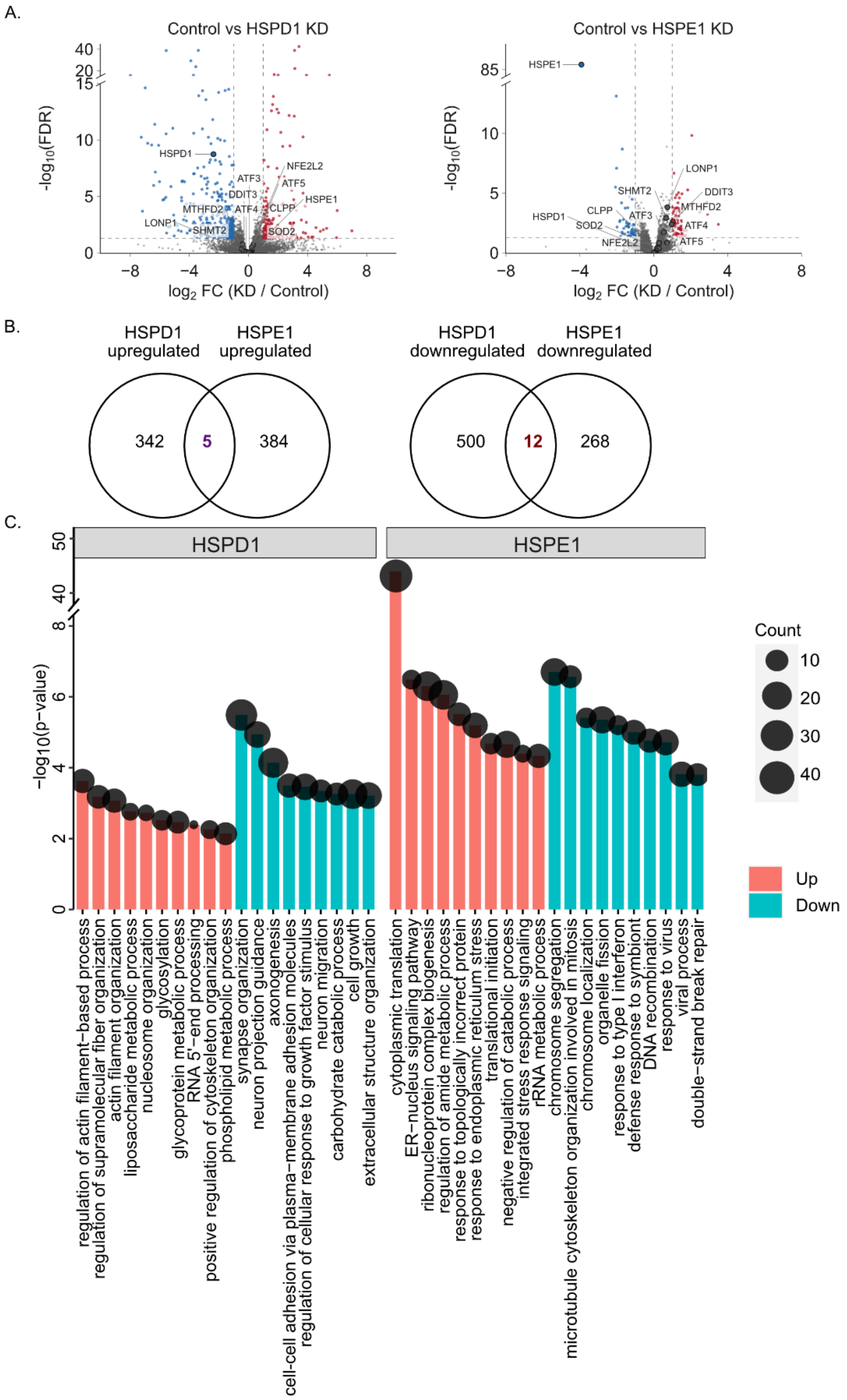

SUP Fig. 1.

A. Gene expression in HSPD1 or HSPE1 KD U251 cells vs control cells was measured using RNAseq (3-4 biological replicates per condition; pooling the two kd cell lines together) and are shown using a volcano plot. Downregulation of HSPD1 and HSPE1 is shown in the volcano plot.

B. Ven diagram of up- or down-regulated transcripts in HSPD1 KD and HSPE1 KD cells.

C. Pathways enriched in transcripts upregulated or downregulated by HSPD1 and HSPE1 KD.

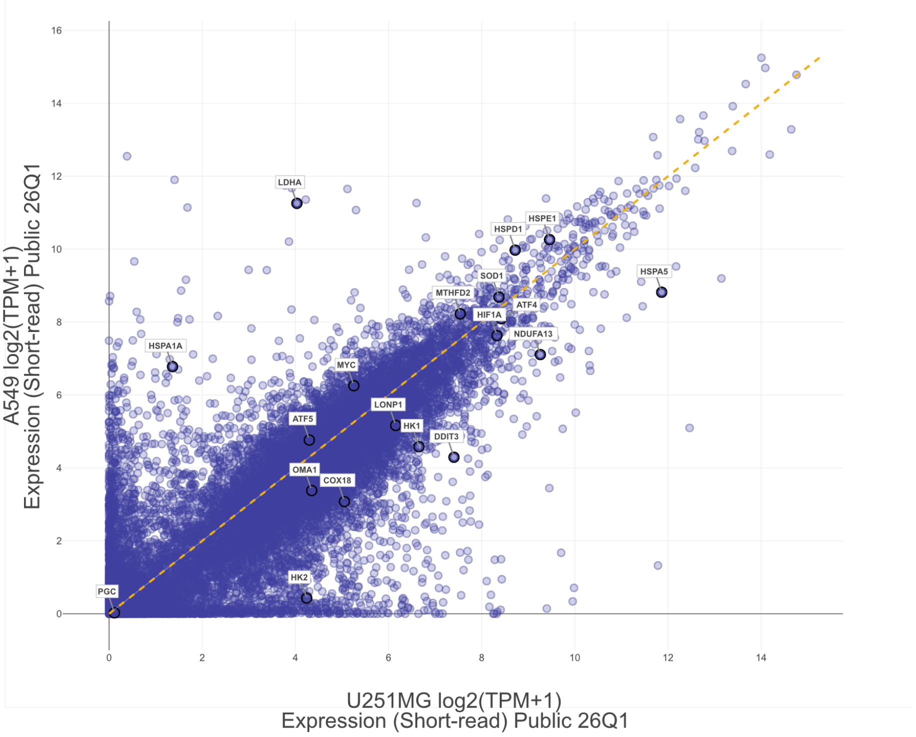

SUP Fig. 2.

Gene expression in A549 and U251 cells was compared using depmap portal (https://depmap.org/)[1,2].

Methods

Cell culture

Human glioblastoma cell line U251 and lung cancer cell line A549 were obtained from ATCC. All cell lines were cultured in Dulbecco's Modified Eagle Medium (DMEM). Medium was supplemented with 10% fetal bovine serum (FBS), sodium pyruvate solution (1mM), and antibiotic–antimycotic (complete DMEM). Cells were kept in an incubator with 5% CO_2 at 37°C.

Generation of stable cell lines for gene knockdown

Gene knockout of HSPD1 was performed using the SYNTHEGO Gene Knockout Kit v2. A549 and U251 cells were seeded into 6-well plates at a density of 200,000 cells per well and allowed to grow overnight. The next day, cells were transiently transfected with HSPD1-specific CRISPR guide RNAs pool (gRNAs) and CAS9 to a final concentration of 3uM, using Lipofectamine Cas9 Plus reagent and following the recommended protocol. HSPD1 protein levels were measured using western blot three days post-transfection, and three "pool-CRISPR-knockdown" populations were analyzed. WT cells were transfected similarly, without CAS9. Next, a single clone isolation protocol was performed to select clones with HSPD1 downregulation. Pool-CRISPR cells were seeded in 96 well plates at a concentration of 0.5 cells well. Filtered DMEM from proliferating U251 WT cells was used in addition to fresh DMEM until small colonies were observed by light microscopy. Once clones filled a well, they were transferred to a larger well plate. Immunoblotting was used to validate the HSPD1 knockdown.

Lentiviral silencing

Two different CRISPR clones were chosen for HSPD1 knockdown using two independent (Short hairpin RNA) shRNA (SIGMA ALDRICH, MISSION shRNA, TRCN0000029446 and TRCN0000343952 for sh_1 and sh_2, respectively). For lentivirus generation, HEK293T Cells were grown in complete DMEM and transfected using CalFectin and a plasmid ratio of 1:2:3 (PAX2; pMD2.G; transfer vector). The media was changed after 24 hours, and the viruses were collected after 48 hours. The collected viruses were stored at -80°C. Prior to virus infection, cells were grown to 60% confluence on 6-well plates and then infected with the virus at a ratio of 1:10 (shCONT, sh#1, sh#2) and left for 24 hours in a 37°C incubator. Following infection, the containing medium was removed, replaced with a fresh medium, and incubated at 37°C. Puromycin (1:1000) was used for selecting infected cells. Gene knockdown was validated using qRT-PCR and Western blot.

Metabolic extraction for liquid chromatography-mass spectrometry (LCMS)

Control and two KD cell lines were seeded in 6cm plates at 37 °C overnight (400,000 cells per well, a minimum of six plates from each cell line). An additional 12-well plate with 100,000 cells per well (four wells for each cell line) was prepared for normalization by crystal violet

staining. The next day, metabolite extraction was performed as follows. First, the media was aspirated, and the cells were washed twice with 1ml of HPLC-grade saline, ensuring complete saline removal before proceeding to the next step. Liquid nitrogen was poured onto the plate and placed in a liquid nitrogen tank. Plates were placed on ice for metabolite extraction. Metabolites were extracted with 1ml of a solution containing 50% methanol, 30% acetonitrile, and 20% water, all of which were of HPLC grade. Next, the plates were placed on an orbital shaker for 10 minutes at 4 °C. The extraction solution from each plate was then transferred into Eppendorf tubes and vortexed. Subsequently, the samples were centrifuged at 16,100g for 10 minutes at 4 °C. After centrifugation, all the supernatants were transferred to HPLC glass vials and were stored at -80 °C prior to LC-MS analysis.

RNA sequencing

Total RNA was extracted from cells using an RNA purification kit (Invitrogen) according to the manufacturer's instructions. RNA concentration and quality were determined using a NanoDrop spectrophotometer. Samples were then sent to the sequencing unit in Ben-Gurion for library preparation and sequencing. RNA integrity was evaluated using a QIAxcel device (QIAGEN, QIAxcel RNA QC Kit v2.0), and RNA concentration was measured using the QuantiFluor® RNA System (Promega, #E3310) on a Qubit™ Flex Fluorometer (Invitrogen). From 0.5 µg of total RNA, mRNA was enriched using the NEBNext® Poly(A) mRNA Magnetic Isolation Module (New England Biolabs, #E7490), ensuring specific capture of polyadenylated transcripts. According to the manufacturer's instructions, stranded RNA-seq libraries were prepared using the NEBNext® Ultra II Directional RNA Library Prep Kit for Illumina® (New England Biolabs, #E7760). The molarity of RNA-seq libraries was determined using the QIAxcel device (QIAxcel DNA High Sensitivity Kit) and the QuantiFluor® dsDNA System (Promega, #E2670) on a Qubit™ Flex Fluorometer (Invitrogen). Libraries were sequenced on a NovaSeq X system (Illumina) with paired-end reads of 150 base pairs (150PE). Sequencing data were processed by Linor Cohen using a bioinformatics pipeline implemented in the software NeatSeq-Flow[3].

Differential Expression Analysis

Gene-level differential expression analysis was performed using the NeatSeq-Flow platform DESeq2[3] module. Expression matrices from RSEM were used as input. The statistical model included a batch as well as genotype and fraction interaction, predefined contrasts tested fraction comparisons within each cell line separately. Genes with fold change ≥1, adjusted P-value <.05 were considered as significantly differentially expressed genes. Significant genes were clustered using the 'eclust' function from the factorextra R package[4]. Enrichment for Gene Ontology biological processes and KEGG pathways was performed using clusterProfiler v4.0 R package[5].

C. elegans strains and maintenance

All *C. elegans* strains used in this work are listed in Table 5. Nematodes were maintained on NGM plates seeded with OP50-1 *Escherichia coli* bacteria at 15 °C. For each experiment,

eggs were picked from plates maintained at 15 °C and transferred to 20 °C for the duration of the experiments.

To induce male formation, synchronized young adult hermaphrodites were placed on fresh NGM plates (5–10 worms per plate) and incubated at 32°C for 4 hours. Following this heat shock, plates were maintained at 20 °C for 3 days to allow recovery and development. Plates were then examined under a dissecting microscope for the presence of males. Male worms were subsequently used for crosses to generate experimental lines and to outcross the CRISPR lines into our N2 (WT) strain. The genotypes of crossed progeny were determined by PCR followed by gel electrophoresis and sequencing.

Live imaging preparation

For live imaging, worms were mounted on 2% agarose pads prepared on glass slides containing 1.5 mM levamisole for immobilization. Approximately 40 µl of agarose was placed on a glass slide and immediately covered with a coverslip to form the pad. Worms were transferred into a 1–2 µl drop of levamisole on the pad, covered with a coverslip, and imaged immediately after preparation to ensure accuracy of cellular structures.

Thrashing Assay (Liquid Motility)

Motility of age-synchronized worms was analyzed. An individual worm was transferred into 100 µL of M9 buffer in a plate and allowed to acclimate for 5 min. Motility was quantified by counting the number of body bends over a 20s interval under a stereomicroscope, and values were converted to body bends per minute. For comparison, CRISPR-generated mutant worms targeting *hsp-60* and *hsp-10* were analyzed, with SJ4100 mtUPR reporter worms used as the control strain.

Table 1. Materials

Material	Source	Identifier
DMEM, high glucose	Gibco	Cat#11965092
Fetal bovine serum (FBS)	ThermoFisher Scientific	Cat#10270106

sodium pyruvate solution	Biological Industries	Cat#03-042-1
antibiotic–antimycotic 100X	TOKU-E	Cat#A045
Gene Knockout Kit v2	SYNTHEGO	Cat#111
Lipofectamine CRISPRMAX Cas9 transfection reagents	ThermoFisher Scientific	Cat#CMAX00001
Opti-MEM I reduced serum medium.	ThermoFisher Scientific	Cat#31985062
HPLC grade water	Sigma-Aldrich	Cat#34877

Table 2. shRNA

shControl (for HSPD1 KD)	CCTAAGGTTAAGTCGCCCTCGCTCGAGCGAGGGCGACTTAACCTTAGG
shHSPD1 #1	CCGGCCTGCTCTTGAAATTGCCAATCTCGAGATTGGCAATTTCAAGAGCAGGTTTTT
shHSPD1 #2	CCGGGCAATGACCATTGCTAAGAATCTCGAGATTCTTAGCAATGGTCATTGCTTTTT
shControl (for	ATCTCGCTTGGGCGAGAGTAAG

HSPE1 KD)	
shHSPE1 #1	AATCTTGGGCTAGGTTGTC
shHSPE1 #2	AATAGAGGTTGAAAGTGCG

Table 3. Plasmids

Plasmid	Source	Identifier
pMD2.G	Addgene	Cat#12259
psPAX2	Addgene	Cat#12260

Table 4. Buffers

Buffer	Component and final concentrations
M9 buffer	22 mM KH_2PO_4, 42 mM Na_2HPO_4, 85 mM NaCl, 1 mM $MgSO_4$

Table 5. Experimental models

Organism/Strain	Source
C. elegans: N2, SJ4100, VH7154, VC4728	CGC, which is funded by NIH Office of Research Infrastructure Programs (P40 OD010440)

References

1. Arafeh, R., Shibue, T., Dempster, J. M., Hahn, W. C. & Vazquez, F. The present and future of the Cancer Dependency Map. *Nat. Rev. Cancer* **25**, 59–73 (2025).

2. DepMap, Broad. DepMap Public 25Q3.

3. Sklarz, M., Levin, L., Gordon, M. & Chalifa-Caspi, V. NeatSeq-Flow: A Lightweight High-Throughput Sequencing Workflow Platform for Non-Programmers and Programmers Alike. *bioRxiv* 173005 (2018) doi:10.1101/173005.

4. Love, M. I., Huber, W. & Anders, S. Moderated estimation of fold change and dispersion for RNA-seq data with DESeq2. *Genome Biol.* **15**, 550 (2014).

5. Yu, G. Thirteen years of clusterProfiler. *The Innovation* **5**, 100722 (2024).
